# Additives in Processed Foods as a Potential Source of Endocrine-Disrupting Chemicals: A Review

**DOI:** 10.3390/jox14040090

**Published:** 2024-11-04

**Authors:** Anand Paramasivam, Rajadurai Murugan, Mathew Jeraud, Angel Dakkumadugula, Ravisankar Periyasamy, Selvam Arjunan

**Affiliations:** 1 Department of Physiology, RVS Dental College and Hospital (Affiliated to The Tamil Nadu Dr. M.G.R. Medical University, Chennai 600032, Tamil Nadu, India), Kumaran Kottam Campus, Kannampalayan, Coimbatore 641402, Tamil Nadu, India; 2 Department of Food Technology, Faculty of Life and Allied Health Sciences, M S Ramaiah University of Applied Sciences, Bangalore 560054, Karnataka, India; 3 Department of Physiology, Ibn Sina National College for Medical Studies, Jeddah 22421, Saudi Arabia; mathewphysio@ibnsina.edu.sa; 4 Clinical Division, Indian Council of Medical Research-National Institute of Nutrition, Hyderabad 500007, Telangana, India; angel2002pinky@gmail.com; 5 Department of Anatomy, SRM Dental College, SRM Institute of Science and Technology, Bharathi Salai, Ramapuram Campus, Chennai 600089, Tamil Nadu, India; ravi_anat@yahoo.com; 6 Lerner Research Institute, Department of Cardiovascular and Metabolic Sciences, Cleveland Clinic, 9500 Euclid Avenue, Cleveland, OH 44195, USA; arjunas@ccf.org

**Keywords:** food additives, endocrine-disrupting chemicals, receptor binding, gene expression, thyroid dysfunction, developmental effects

## Abstract

Processed foods, accounting for most consumable food categories today, contain considerable amounts of food additives. Food additives are substances added to food products to improve taste, consistency, appearance, or shelf life. Various food additives, such as phthalates, bisphenol A, tartrazine, erythrosine, artificial sweeteners, and parabens, have been identified as potential sources of endocrine-disrupting chemicals (EDCs) in processed foods. EDCs are substances that frequently interfere with the regular functioning of the endocrine system, creating an unusual environment in the biological system, which leads to adverse health effects such as the disruption of hormone synthesis, receptor binding, and signal transduction pathways, as well as energy metabolic homeostatic disorders which potentially increasing the risk of obesity, type-2 diabetes, cardiometabolic diseases and may also trigger allergic reactions. Consequently, they can also impact mammary gland development, and reproductive function, further leading to developmental abnormalities. This review aims to insights into the various food additives that act as potential endocrine-disrupting chemicals (EDCs) and to describe their applications in the food industry, as well as the failure of hormonal homeostatic mechanisms, which eventually result in hazardous health effects. It also outlines strategies to reduce the use of food additives and suggests alternative additives with minimal or no endocrine-disrupting properties, highlighting their importance for maintaining human health.

## 1. Introduction

According to the *Codex Alimentarius*, food additives are substances that are not typically eaten as food by themselves and are not usually employed as ingredients in food. However, they are intentionally added to enhance the organoleptic properties of foods during the production, processing, preparation, packaging, and transportation of food to serve a specific purpose, such as improving the color, appearance, flavor, taste, or texture. Using food additives helps improve the quality of the final product and extends the shelf life of food on store shelves [1]. The food industry can only use food additives if necessary for specific reasons, where they cannot be replaced by other substances that are both economically and technically feasible. Additionally, these additives must not pose any hazardous effects on consumer health when used within the recommended levels [1,2].

There are several types of food additives, including nutritional additives (these are added to food to make it more nutritious, like vitamins that act as antioxidants, amino acids, and bioelements), flavoring agents (used to provide or enhance a specific taste in food), texturizing agents (substances that help stabilize and emulsify food), and coloring agents (these additives are used to make food products look more visually appealing) [3]. EDCs are substances that come from outside the body and interfere with the natural hormones, hormone receptors, or receptors of neurotransmitters that are responsible for keeping our bodies healthy and balanced, helping with reproduction, and supporting the overall growth and development of the body.

Diamanti et al., in 2009, reported that EDCs affect certain receptors, such as estrogen receptors (ERs), androgen receptors (ARs), progesterone receptors (PRs), thyroid receptors (TRs), and retinoid receptors (RRs) [4]. EDCs affect different parts of the body by interacting with specific receptors, including nuclear and cell membrane receptors. These disruptors can also influence the enzymes involved in the synthesis and breakdown of hormones, along with other mechanisms that affect the endocrine and reproductive systems. They can also interact with receptors that are unrelated to hormones, such as receptors for neurotransmitters like serotonin, dopamine, and norepinephrine [4,5].

Generally, food additives are classified into intentional and unintentional food additives. Food additives are added to processed and packaged food items not only during preparation or processing but also during packaging with various packaging materials. This review focuses on selected food additives that can disrupt the endocrine system, particularly in processed foods. This precise approach was selected due to the increased consumption of processed and packaged food items available today. We also explore how EDCs disrupt the endocrine system, cause hormonal imbalances, and exert various hazardous effects on the biological system. Similarly, we consider how to reduce the usage of food additives and suggest alternative additives with fewer or no endocrine-disrupting properties.

## 2. Methodology

The required data were searched and collected online, including previously published data in journals, textbooks, periodicals, websites, and databases such as Wiley, Google, PubMed, Google Scholar, Science Direct, Web of Science, SIIC Data Bases, BIOSIS, CAB International, Cambridge Scientific Abstracts, Chemical Abstracts, Food Science and Technology Abstracts, Research Alert, Science Citation Index, Science Citation Index Expanded, and Scopus-indexed journals, as well as other online sources. The keywords used in this search included food additives, endocrine-disrupting chemicals, receptor binding, gene expression, thyroid dysfunction, developmental effects, intentional food additives, and unintentional food additives [6]. The inclusion criteria for articles are as follows: 1. The articles must be scientific and focus on food additives and endocrine-disrupting chemicals (EDCs). 2. Food additives should be documented as endocrine disruptors in scientific literature. 3. Studies should involve human or animal exposure to these food additives. 4. Research must demonstrate a biologically credible mechanism of endocrine disruption. 5. There should be a clear link between the EDCs and endocrine-related health effects. Exclusion criteria for articles included food additives not classified as endocrine disruptors by any recognized authority, studies lacking sufficient data on endocrine activity, and research focusing on non-endocrine-related health effects.

## 3. Sources of EDCs Entering the Body

Several EDCs enter into the body through various ways, including plastics, perfumes, fragrances, water sources, pesticides, and food additives. Even though food additives are a major source of endocrine disruptors, some other substances associated with food items also act as EDCs. These include plastics in food packaging materials, pesticides from edible crops, and food additives in processed foods.

### 3.1. EDCs from Food Packaging Materials and Pesticides from Agro Products

Plastics are a significant source of EDC, which includes substances from plastics like bisphenol A (BPA), which can leach out of plastics, particularly when they are heated. Once released, BPA can be ingested or absorbed through the skin, leading to exposure in humans [7]. Other EDCs that can be found in plastics include phthalates. Several classes of pesticides have been identified as EDCs, including organophosphates, organochlorines, and pyrethroids. These pesticides can contaminate food through various pathways, including residues on crops, soil, and water sources used in agriculture [8]. To minimize exposure to EDC-containing pesticides in food, regulatory agencies establish maximum residue limits and safety standards for pesticide use [9].

EDCs in plastics and pesticides are a concern because these compounds can interfere with the endocrine system, disrupting hormonal balance and affecting normal physiological processes. EDCs from plastics and pesticides can mimic or alter the functions of natural hormones, leading to a range of adverse effects, including reproductive health, development, immune function, metabolism, and other hormone-regulated biochemical reactions in both humans and wildlife [10].

### 3.2. EDCs from Processed Foods as Additives

Endocrine-disrupting chemicals can also be found in processed foods as additives. These additives are added to processed foods to enhance flavor, improve texture, prolong the shelf life, and add or improve color. Some of these additives have been identified as EDCs and can potentially disrupt the normal functioning of the endocrine system when consumed [11]. Examples of EDCs found in processed foods include certain food dyes, preservatives, and plasticizers. Food dyes, such as Red 3 (erythrosine) and Yellow 5 (tartrazine), have been associated with endocrine-disrupting effects in animal studies. These dyes can mimic or interfere with hormone signaling, leading to potential disruptions in hormonal balance [11,12]. Parabens are even used as a preservative in processed food. However, these additives have been shown to exhibit endocrine-disrupting properties, potentially interfering with hormone regulation and contributing to adverse health effects. Long-term exposure to these compounds, particularly at high levels, may increase the risk of endocrine-related disorders, reproductive problems, and other adverse health outcomes [12] (Table 1).

## 4. Additives Present in Processed Foods That Act as Major EDCs

This review focuses on how food additives act as EDCs, their sources, usage in food industries, mechanism of action, and health hazards. The following food additives and their endocrine-disrupting properties are specifically examined. Food additives acting as EDCs include [13].

- Red 3 (erythrosine);
- Yellow 5 (tartrazine);
- Bisphenol A (BPA);
- Artificial sweeteners;
- Phthalates;
- Parabens.

### 4.1. Red No. 3 (Erythrosine): Sources, Applications, Mechanism of Action, and Health Hazards

Red No. 3 is derived from coal tar, a byproduct of coal processing that contains various aromatic compounds. One of these compounds is called fluoranthene, which serves as the starting material for the synthesis of erythrosine [13]. Erythrosine has been used to provide a vibrant red color to candies, icings, frostings, gelatins, and other sweet treats. It has been used in red velvet cake, strawberry-flavored desserts, and fruit fillings. It has been employed as a coloring agent in certain beverages, such as fruit-flavored drinks, soda pops, and cocktail mixers, to give a visually appealing red shade. Furthermore, it is added to processed meat products, such as sausages, to enhance their appearance and used to add a red shade to dairy products, such as yogurts, ice creams, and milkshakes [8,13]. Erythrosine has also found applications beyond the food industry, like a coloring agent in pharmaceutical preparations, including tablets and capsules, and cosmetics, such as lipsticks and blushes, to impart a red color [13].

#### 4.1.1. Mechanism of Action of Erythrosine as EDC

**Figure 1:**
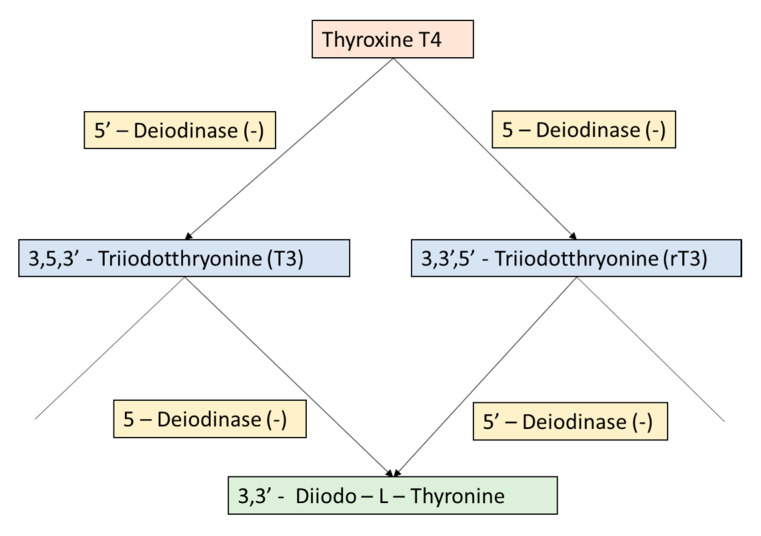
The effects of erythrosine on the in vivo metabolism of thyroxine by inhibiting the 5 o-deoidinase that normally converts T4 and T3 (Capen et al., 1989) [14].

The ingestion of erythrosine via processed food can interfere with the uptake of iodine by the thyroid gland and cause decreased thyroid hormone synthesis and secretion (Figure 1).

#### 4.1.2. Endocrine Disruption

Erythrosine disrupts the production of hormones by affecting the function of endocrine glands, such as the thyroid gland or the adrenal glands, which leads to imbalances in hormone levels and disturbs the normal regulation of physiological processes [15]. Erythrosine has been reported to exhibit estrogenic activity in some studies, meaning it can mimic or modulate the effects of estrogen in the body. This estrogenic activity can disrupt normal hormonal balance and impact reproductive processes and other estrogen-dependent functions. Conversely, erythrosine has also been found to have anti-estrogenic effects in certain experimental settings, further highlighting its potential to interfere with hormone signaling [15,16].

#### 4.1.3. Thyroid Dysfunction

The thyroid gland is crucial in regulating metabolism, growth, and development. Erythrosine has been shown to affect the binding of thyroid hormones to their receptors, which are necessary for normal thyroid hormone signaling. By interfering with this process, erythrosine may disrupt the regulation of gene expression and other physiological processes controlled by thyroid hormones [15,16]. Specifically, erythrosine action is associated with decreased levels of T3 and T4 and increased levels of thyroid-stimulating hormone (TSH), which may indicate thyroid dysfunction or an adaptive response to erythrosine exposure [16].

#### 4.1.4. Red No. 3 Inducing Cancer

There is even a potential link between erythrosine exposure and the development of tumors, particularly in the thyroid and adrenal glands. Erythrosine has been shown to have genotoxic effects in certain studies and can cause DNA damage. The genotoxic effects of erythrosine may be related to its potential to produce reactive oxygen species (ROS) and induce oxidative stress, which can lead to DNA damage and potentially initiate or promote cancer formation [16]. Erythrosine can even stimulate cell proliferation in certain cell types. It is well known that increased cell proliferation can contribute to the formation of tumors and the progression of cancer. Erythrosine and EDCs have the potential to interfere with hormone regulation and signaling, which could be the reason for the development of hormone-related cancers like breast and prostate cancer [16,17].

#### 4.1.5. Role of Red No. 3 in Inducing Allergic Response

Repeated exposure to erythrosine may lead to sensitization of the immune system. The immune system becomes hypersensitive to erythrosine and reacts excessively upon subsequent exposures. Sensitization can result in the production of specific antibodies called immunoglobulin E [IgE] antibodies, which can trigger allergic reactions. Cross-reactivity occurs when the immune system recognizes similar structural features in different substances and reacts to them. Individuals who are allergic to other food dyes or related compounds may also experience allergic reactions to erythrosine due to cross-reactivity. Genetic factors, previous allergic sensitization, and individual immune system characteristics can contribute to the susceptibility to allergic reactions [16,17].

### 4.2. Yellow No. 5, Tartrazine: Sources, Applications, Mechanism of Action, and Health Hazards

Tartrazine is derived from coal tar, which is a byproduct of coal processing and contains various aromatic compounds. One of these compounds, called benzoic acid, serves as the starting material for the synthesis of tartrazine [18]. Yellow No. 5 (tartrazine) is widely used in food industries to provide a bright yellow color, often used to give a vibrant yellow color to fruit juices, soft drinks, energy drinks, sports drinks, powdered drink mixes, and flavored water. It is also used in candies; chewing gum; gummy bears; jellybeans; other confectionery products; some sauces, including mustard and salad dressings; pickles; etc. Tartrazine is found in custards, puddings, ice creams, sorbets, and other sweet treats to provide a yellow coloring [18].

#### 4.2.1. Mechanism of Action of Yellow No. 5 [Tartrazine]

Tartrazine can potentially bind to hormone receptors, including estrogen or other hormone receptors, and either mimic or block the actions of natural hormones. Tartrazine, by binding with estrogen receptors, induces estrogenic responses in certain cell-based assays [19]. Tartrazine’s interaction with estrogen receptors may alter gene expression patterns. Binding to estrogen receptors can activate or inhibit specific genes, potentially influencing cellular functions and hormone-related processes [19,20].

#### 4.2.2. Hormone Synthesis and Metabolism

Tartrazine might influence the activity of enzymes involved in hormone synthesis and metabolism. Tartrazine can potentially modulate the activity of enzymes involved in hormone biosynthesis, leading to alterations in hormone synthesis or clearance. It might affect the availability of hormone precursors, which are necessary for the synthesis of specific hormones. By interfering with the availability or utilization of precursors, tartrazine could indirectly impact hormone synthesis [19,20]. Tartrazine can potentially influence the function of endocrine glands, such as the thyroid and adrenal glands, which leads to alterations in hormone production and metabolism. Hormone synthesis and metabolism are tightly regulated by feedback mechanisms within the endocrine system. Tartrazine may interfere with these feedback loops, disrupting the normal regulation of hormone production and metabolism [20].

#### 4.2.3. Altering Gene Expression

Epigenetic changes are the alterations in gene expression that do not include modifications in the underlying DNA sequence. Tartrazine may have the ability to induce epigenetic modifications, such as DNA methylation or histone modifications, which can cause changes in gene expression patterns [21]. Tartrazine might interact with transcription factors, proteins that attach to DNA and influence the expression of specific genes. By interacting with these transcription factors, tartrazine could potentially influence the activation or inhibition of target genes, which affects the signaling pathways involved in gene expression regulation. These pathways transmit signals from the cell surface to the nucleus, activating or inhibiting specific genes. As an EDC, tartrazine may interfere with hormone signaling pathways, which could indirectly impact gene expression patterns [21,22].

### 4.3. Bisphenol A [BPA]: Sources, Applications, Mechanism of Action, and Health Hazards

#### 4.3.1. Sources and Applications

**Figure 2:**
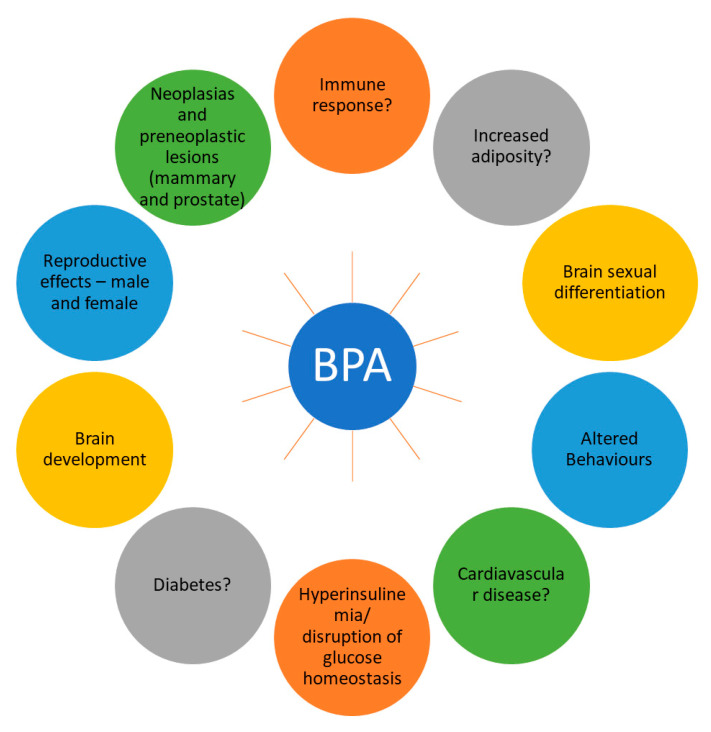
The effects of Bisphenol A as an EDC on various diseases/disorders (Okugbe et al., 2019) [24].

Bisphenol A (BPA) is also used in the production of epoxy resins, which are widely employed as coatings on the interior of metal food and beverage cans to prevent corrosion and maintain the quality of the contents, which are clear, hard, and durable [23]. These plastics have been used to make food storage containers, water bottles, and other food-related plastic items in water-dispensing equipment [23]. BPA is not directly used as a food additive, which is an unintentional food additive. However, it is used in certain food-related applications due to its protection of the food or beverage from directly contacting the metal. This coating helps prevent corrosion and maintain the quality of the product (Figure 2).

#### 4.3.2. Effects of Bisphenol A on Estrogenic Activity

BPA can mimic or modulate estrogen activities, a natural hormone in the body. It can bind to estrogen receptors, activate them, and exert estrogen-like effects. BPA’s interaction with estrogen receptors can disrupt normal hormonal signaling and alter various physiological processes regulated by estrogen. BPA can adhere to estrogen receptor alpha (ERα) and estrogen receptor beta (ERβ), which are proteins located within cells that mediate the effects of estrogen. By binding to these receptors, BPA can activate them and initiate estrogen-like signaling pathways. When BPA binds to estrogen receptors, it can induce the expression of certain genes that are regulated by estrogen [24,25]. This can result in the activation of genes involved in various physiological processes, including cell growth, development, and hormone production. This can lead to hormone-like effects, such as stimulating the growth of estrogen-sensitive tissues, promoting the proliferation of cells, and influencing hormone-dependent pathways [24,25].

#### 4.3.3. Bisphenol A in Hormone Signaling Disruption

Bisphenol A can disrupt the production and release of hormones from the ovaries, testes, and adrenal glands, affecting the overall hormonal balance in the body [25,26]. It has also been found to affect thyroid hormone signaling. It can inhibit the production of thyroid hormones, alter their transport proteins, and disrupt the binding of thyroid hormones on their receptors, causing disturbances in thyroid hormone function, which is critical for growth, development, and metabolism [26]. BPA can also interact with androgen receptors (ARs), which are involved in mediating the effects of androgens, such as testosterone. BPA’s interaction with ARs can disrupt androgen signaling and potentially affect processes regulated by androgens, including sexual development and reproductive functions [26].

#### 4.3.4. Gene Expression Alteration

BPA exposure has been associated with changes in epigenetic modifications, which are chemical modifications to the DNA or its adjunct proteins that can control gene expression without changing the DNA sequence. Such changes include DNA methylation, histone modifications, and changes in microRNA expression [26,27]. It can affect the transcriptional activity of genes, which refers to the process of converting DNA into RNA. BPA can influence the binding of transcription factors that control the gene expression process with specific gene-regulatory regions, thus modulating the expression of target genes. These alterations can lead to developmental effects, affecting the normal development and function of organs and overall biological systems [26,27].

#### 4.3.5. Developmental Effects

BPA exposure has been linked to alterations in the development and function of the reproductive system. It can affect fertility, hormone production, and reproductive behaviors. Furthermore, animal studies have shown that BPA can influence brain development, leading to changes in behavior, learning, memory, and social interactions. Prenatal and early life exposure to BPA has been associated with metabolic effects, including elevated body mass, altered fat metabolism, and insulin resistance. BPA exposure may contribute to a greater risk of obesity, type-2 diabetes, and metabolic syndrome later in life [27]. Additionally, BPA exposure has been associated with changes in mammary gland development, which can affect susceptibility to breast cancer later in life [27].

### 4.4. Artificial Sweeteners: Sources, Applications, Mechanism of Action, and Health Hazards

The basic process of preparing artificial sweeteners involves synthesizing compounds with sweet taste properties and modifying their molecular structure to enhance the sweetness and other desired characteristics rather than obtaining them naturally [28]. They are commonly used in sugar-free products, including carbonated drinks, juices, flavored waters, and powdered drink mixes, and in the production of low-calorie or reduced-calorie foods, such as desserts, yogurts, ice creams, and snack bars, to provide sweetness without adding significant calories. Additionally, artificial sweeteners can be used to formulate pharmaceutical products, such as syrups and liquid medications, to mask the bitter taste of active ingredients. Some artificial sweeteners are used in toothpaste, mouthwash, and other oral care products to enhance flavor without contributing to tooth decay [28].

#### 4.4.1. Mechanism of Action of Artificial Sweeteners

Artificial sweeteners (ASs) show EDC properties in the following ways, including receptor-binding capacity, interfering and modulating the actions of the gut microbiota, and influencing glucose metabolism.

#### 4.4.2. Modulation of Hormones and Gut Microbiota

Artificial sweeteners, being non-caloric or low-caloric, do not provide nutrients for the gut bacteria [29]. Artificial sweeteners like aspartame and acesulfame potassium have been suggested to influence the release and activity of certain hormones, such as insulin, leptin, and ghrelin, which are involved in appetite regulation [30]. ASs may influence the composition and function of the gut microbiota, which plays a vivid role in various aspects of health, including metabolism and hormonal regulation. ASs influence the gut microbiota and could potentially impact the production and metabolism of hormones, which directly and indirectly act as EDCs [30]. They also have been shown to affect host metabolism and can influence factors such as glucose regulation and insulin sensitivity. These changes in host metabolism can, in turn, indirectly influence the gut environment and the composition of the gut microbiota [29].

### 4.5. Phthalates: Sources, Applications, Mechanism of Action, and Health Hazards

**Figure 3:**
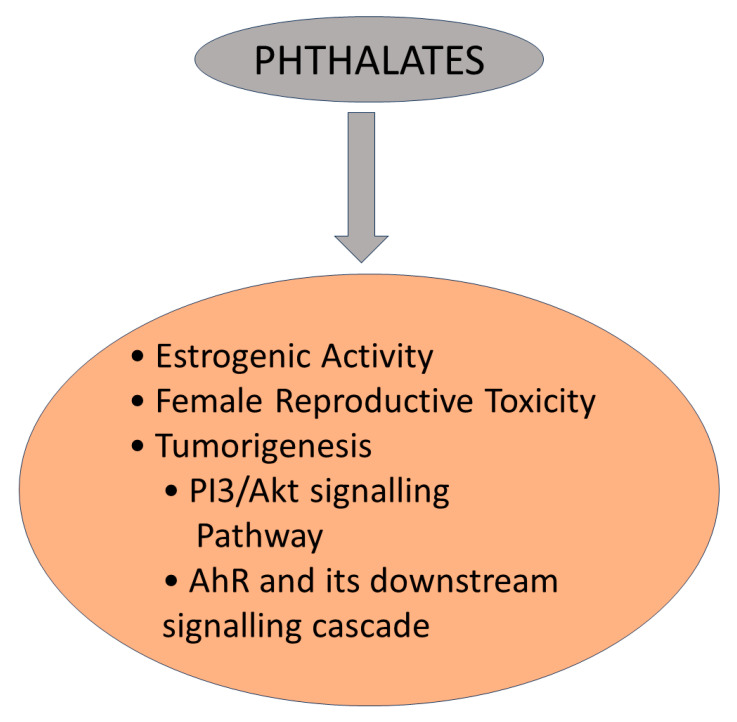
The mechanism of action of phthalates as an EDC (Del et al., 2016) [33].

The production of phthalates involves a chemical reaction between phthalic anhydride, derived from the oxidation of naphthalene, in which alcohol used in the reaction determines the type of phthalate produced. For example, diethyl phthalate is produced by reacting phthalic anhydride with ethanol [31]. Phthalates are not intentionally used as additives in processed foods. However, these contaminants can enter food through various routes, such as migration from packaging materials or contamination during processing, storage, or transportation. Phthalates may be found in trace amounts in certain processed foods due to their presence in packaging materials, including plastic food containers, wraps, and films [32]. They can migrate from these materials into the food, especially under certain conditions, such as high temperatures or prolonged storage. Certain phthalates may have endocrine-disrupting properties and could interfere with hormone signaling in the body [31] (Figure 3).

#### 4.5.1. Binding to Hormone Receptors

Phthalates can interact with hormone receptors, including estrogen receptors (ERs), androgen receptors (ARs), and peroxisome proliferator-activated receptors (PPARs). By binding to these receptors, phthalates can mimic or completely stop the activities of natural hormones, causing alterations in hormone signaling pathways. Some phthalates have been recognized to bind to ER, and they can act like estrogen mimics (bind to estrogen receptors and exert estrogenic effects in the body). This can disrupt normal estrogen signaling and affect hormone balance [34,35]. Certain phthalates, including butylbenzylphthalate and diisononyl phthalate, have been shown to interact with androgen receptors; they can exhibit anti-androgenic effects, competing with natural androgens (such as testosterone) for binding to androgen receptors, leading to adverse effects on reproductive function and development [34,35].

#### 4.5.2. Disruption of Hormone Synthesis and Metabolism

Phthalates have been found to affect enzymes involved in hormone synthesis and metabolism. They can interfere with the activity of enzymes such as aromatase, which converts androgens to estrogens, or 5-alpha reductase, which converts testosterone to dihydrotestosterone (DHT). These disruptions can lead to imbalances in hormone levels. Phthalates can also affect the metabolism of hormones in the body [34,36]. They have the potential to compete with natural hormones for binding to carrier proteins in the bloodstream, such as sex hormone-binding globulin (SHBG). By displacing hormones from these binding proteins, phthalates can impact hormone availability and alter hormone signaling [34,35].

#### 4.5.3. Alteration of Hormone Transport and Binding Proteins

Phthalates can impact hormone transport and binding proteins in the bloodstream. For example, some phthalates have been shown to displace natural hormones from binding proteins like SHBG, which can affect hormone availability and activity [34,35]. Phthalates can disrupt the interaction between hormones and their specific receptors by altering the availability and concentration of hormones; phthalates may also influence the activation and signaling pathways mediated by hormone receptors, such as ER, AR, and PPARs [35,37].

#### 4.5.4. Disruption of Hormone Signaling Pathways

Phthalates can interfere with hormone signaling pathways by affecting gene expression, signal transduction, and cellular responses to hormones. They may influence the expression of genes that take part in hormone synthesis, metabolism, or receptor activity, leading to changes in hormone responsiveness [35,37]. They can affect the expression of hormone-responsive genes, altering the production of proteins involved in hormone synthesis, metabolism, and signaling. These changes in gene expression can lead to imbalances in hormone levels and perturb normal hormone signaling pathways. Hormone signaling is tightly regulated through feedback mechanisms that maintain hormone balance in the body. Phthalates can disrupt these feedback mechanisms by altering hormone synthesis, metabolism, or receptor interactions [35,37].

### 4.6. Parabens: Sources, Applications, Mechanism of Action, and Health Hazards

Parabens are synthetic compounds that are derived from para-hydroxybenzoic acid. They are produced by esterification of para-hydroxybenzoic acid with an alcohol, typically methanol or ethyl alcohol [38]. Parabens are used as preservatives in certain processed foods to inhibit microbial growth and extend their shelf life. Parabens may be used in certain sauces and dressings to prevent spoilage and maintain product quality. They can be found in baked goods, pastries, and confectionery items to inhibit the growth of mold and other microorganisms and are used in certain beverages, such as fruit juices, soft drinks, and alcoholic beverages, to extend their shelf life by preventing microbial contamination [38].

#### 4.6.1. Mechanism of Action of Parabens as EDC

**Figure 4:**
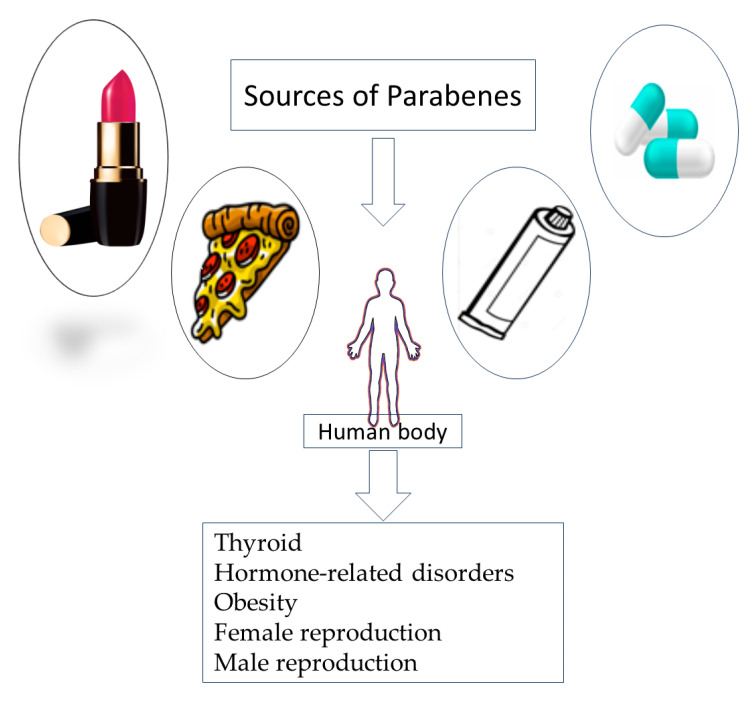
The mechanism of action of parabens as an EDC (Karolina et al., 2018) [39].

Parabens inhibit the activity of enzymes such as aromatase, which converts androgens to estrogens or affects other enzymes involved in hormone metabolism. These alterations in hormone synthesis and metabolism can disrupt the normal production and breakdown of hormones in the body (Figure 4).

#### 4.6.2. Estrogenic Activity and Other Metabolic Actions

Parabens elicit weak estrogenic activity; they can bind to ER and mimic the effects of estrogens. This can potentially lead to disruptions in hormonal balance and interfere with normal estrogen-signaling pathways [40]. Particularly, longer-chain parabens may inhibit aromatase activity, leading to reduced estrogen production. They also have been shown to interfere with the function of thyroid peroxidase, hence affecting the production of thyroid hormones, which are crucial for conducting metabolism and development [40,41,42].

#### 4.6.3. Disruption of Hormone Transport and Binding

SHBG is a protein that binds to sex hormones, such as estrogen and testosterone, in the bloodstream, regulating their availability and activity. By interacting with SHBG and other hormone-transport proteins, parabens may affect the transport, distribution, and bioavailability of hormones in the body [41,42,43]. Parabens have been reported to interfere with hormone transport and binding proteins, such as SHBG. By interacting with these proteins, parabens can potentially affect the availability and distribution of hormones in the body [40,44].

## 5. Conclusions and Recommendations

In this review, we summarize the sources, identify food additives as significant EDCs, and their mechanisms of action, and discuss their health hazards. Exposure to EDCs often accrues and resists degradation due to their complex structure, which increases the risk of hormonal disorders and diminishes the quality of life. Additives such as phthalates, bisphenols, artificial dyes, and sweeteners are commonly used in processed foods. These EDCs can disrupt normal endocrine function, leading to reproductive issues, developmental abnormalities, metabolic disorders, and an increased risk of hormone-related issues. Advancing the science of endocrine-disrupting chemicals (EDCs) is essential for protecting our health and environment. Consumers can contribute to minimizing their exposure to EDCs by making informed choices and opting for minimally processed and organic food options. Additionally, raising awareness about the potential risks associated with EDCs in processed food additives is crucial for empowering individuals to make healthier dietary decisions. Consumers can obtain more information about the food by reading the label. By law, food manufacturers must list all direct food additives (natural and artificial). This will create awareness of different names and terms and misbranding. Some food additives have more than one name, which could be regularized by the authorized agencies. It is important to develop and implement prevention and protection strategies for both individuals and policymakers. Key recommendations include prioritizing whole, minimally processed foods, staying hydrated, and adopting a balanced mindset rather than an all-or-nothing approach.

## Figures and Tables

**Table 1:** The composition percentage of additives in different food commodities (Lorenzoni et al., 2012) [5].

Additives	Cereals and Cereal Products (%)	Dairy and Meat Products (%)	Candy and Chocolate (%)	Beverages (%)	Total (o/o)
Flavorings	62.79	37.85	94.42	88.89	78.85
Emulsifiers and stabilizers	41.09	73.56	89.24	44.44	70.73
Acids	20.93	29.85	28.69	94.44	31.62
Natural coloring	37.21	11.85	8.37	63.89	20.94
Artificial coloring	10.08	14.00	28.29	19.44	20.94
Chemical leavening agents	29.46	-	14.34	-	15.81
Preservatives	10.08	22.0	-	75.00	11.11
Anti-caking agents	18.60	2.00	-	-	5.34
Flavor enhancers	16.28	6	-	-	5.13
Humectants	6.98	-	4.38	-	4.27
Sweeteners	-	4.00	3.59	19.44	3.85
Gelling agents	-	-	6.37	-	3.42
Antioxidants	0.78	7.56	-	19.44	2.56
Thickeners	0.78	1.85	1.2	5.56	1.5
Glazing agents	-	2.00	-	-	0.21

## Data Availability

No new data were created or analyzed in this study.
